# Initial anticoagulation strategy for extracorporeal cardiopulmonary resuscitation patients

**DOI:** 10.1186/cc13678

**Published:** 2014-03-17

**Authors:** Y Iwashita, M Matsuduki, M Yukimitsu, K Yokoyama, T Nakata, A Yamamoto, K Ishikura, H Imai

**Affiliations:** 1 Mie University Hospital, Tsu, Mie, Japan

## Introduction

Extracorporeal cardiopulmonary resuscitation (ECPR) is increasingly being used in emergency and critical care medicine in Japan. Although a major complication of this procedure is bleeding, the optimal heparin dose and activated coagulation time (ACT) remain unknown.

## Methods

We retrospectively evaluated the initial heparin doses, ACT values, and complications of patients who received ECPR between February 2011 and November 2013 at the Emergency and Critical Care Center, Mie University Hospital, Japan.

## Results

ECPR was performed in 45 patients, and the ACT was evaluated in 32 patients. All patients were administered 3,000 U unfractionated heparin at the time of priming the circuit. Patients for whom cannulation took a longer time received an additional 2,000 to 3,000 U unfractionated heparin. The average heparin dose administered was 53.6 U/kg body weight. The average ACT was 231.3 seconds. In 17 of the 32 patients, the ACT exceeded 200 seconds. Three patients experienced fatal bleeding in the chest wall, which could not be stabilized by conservative treatment. One patient developed a cerebral infarction. There were no significant differences between the patients with fatal bleeding and those without fatal bleeding with regard to the heparin dose, ACT, and duration of CPR.

## Conclusion

According to the Extracorporeal Life Support Organization guidelines, the target ACT should be around 1.5 times the normal ACT. However, it is difficult to obtain the normal ACT in emergency situations. Many of our patients' ACTs exceeded 200 seconds, and three patients experienced fatal bleeding that was possibly due to chest compression. Post-cardiac arrest patients often experience coagulopathy due to either cardiac arrest or hypothermia therapy. Therefore, an anticoagulation protocol other than the pulmonary extracorporeal membrane oxygenation protocol is required for ECPR patients. We evaluated our anticoagulation protocol for ECPR and observed that our patients' ACTs frequently exceeded the target value and some experienced fatal bleeding. The anticoagulation protocol for post-CPR patients may need to be reconsidered.

